# Lung Stereotactic Body Radiotherapy (SBRT) Using Spot-Scanning Proton Arc (SPArc) Therapy: A Feasibility Study

**DOI:** 10.3389/fonc.2021.664455

**Published:** 2021-04-22

**Authors:** Gang Liu, Lewei Zhao, An Qin, Inga Grills, Rohan Deraniyagala, Craig Stevens, Sheng Zhang, Di Yan, Xiaoqiang Li, Xuanfeng Ding

**Affiliations:** ^1^ Cancer Center, Union Hospital, Tongji Medical College, Huazhong University of Science and Technology, Wuhan, China; ^2^ Department of Radiation Oncology, Beaumont Health System, Royal Oak, MI, United States

**Keywords:** lung, stereotactic body radiation therapy, spot-scanning, proton arc therapy, interplay effect

## Abstract

**Purpose:**

We developed a 4D interplay effect model to quantitatively evaluate breathing-induced interplay effects and assess the feasibility of utilizing spot-scanning proton arc (SPArc) therapy for hypo-fractionated lung stereotactic body radiotherapy (SBRT). The model was then validated by retrospective application to clinical cases.

**Materials and Methods:**

A digital lung 4DCT phantoms was used to mimic targets in diameter of 3cm with breathing motion amplitudes: 5, 10, 15, and 20 mm, respectively. Two planning groups based on robust optimization were generated: (1) Two-field Intensity Modulated Proton Therapy (IMPT) plans and (2) SPArc plans *via* a partial arc. 5,000 cGy relative biological effectiveness (RBE) was prescribed to the internal target volume (ITV) in five fractions. To quantitatively assess the breathing induced interplay effect, the 4D dynamic dose was calculated by synchronizing the breathing pattern with the simulated proton machine delivery sequence, including IMPT, Volumetric repainting (IMPT_volumetric_), iso-layered repainting (IMPT_layer_) and SPArc. Ten lung patients’ 4DCT previously treated with VMAT SBRT, were used to validate the digital lung tumor model. Normal tissue complicated probability (NTCP) of chestwall toxicity was calculated.

**Result:**

Target dose were degraded as the tumor motion amplitude increased. The 4D interplay effect phantom model indicated that motion mitigation effectiveness using SPArc was about five times of IMPT_volumetric_ or IMPT_layer_ using maximum MU/spot as 0.5 MU at 20 mm motion amplitude. The retrospective study showed that SPArc has an advantage in normal tissue sparing. The probability of chestwall’s toxicity were significantly improved from 40.2 ± 29.0% (VMAT) (p = 0.01) and 16.3 ± 12.0% (IMPT) (p = 0.01) to 10.1 ± 5.4% (SPArc). SPArc could play a significant role in the interplay effect mitigation with breathing-induced motion more than 20 mm, where the target D99 of 4D dynamic dose for patient #10 was improved from 4,514 ± 138 cGy [RBE] (IMPT) vs. 4,755 ± 129 cGy [RBE] (SPArc) (p = 0.01).

**Conclusion:**

SPArc effectively mitigated the interplay effect for proton lung SBRT compared to IMPT with repainting and was associated with normal tissue sparing. This technology may make delivery of proton SBRT more technically feasible and less complex with fewer concerns over underdosing the target compared to other proton therapy techniques.

## Introduction

Lung cancer remains a leading cause of cancer mortality in the world (1). Compared to conventional radiotherapy, hypo-fractionated stereotactic body radiotherapy (SBRT) has been proved to improve local tumor control and survival rate for stage I non-small cell lung cancer (NSCLC) patients (2–6). Taking advantage of the unique beam characteristics, Bragg Peak, proton beam therapy could offer a superior dose distribution compared to photon radiotherapy technique in treating locally advanced lung cancer (7). Recently, with the development of pencil beam scanning (PBS) technology, intensity modulated proton therapy (IMPT) offers the potentials to spare the adjacent normal tissues further while maintaining similar or superior target coverage in a more efficient way without using beam specific blocks or compensators compared to passive scatter proton therapy (PSPT) (8–11). However, such scanning technique is susceptible to the interplay effect between proton spot scanning and respiratory induced motion during dose delivery. It eventually leads to an inaccurate dose delivery such as underdose targe or overdoses of the healthy tissue during lung cancer treatment (12, 13). Several motion management strategies were introduced to mitigate the interplay effect, such as repainting, gating, and tracking (14–16), in which volumetric or layer repainting technique has been widely adopted by the proton clinic. With volumetric repainting, the dose delivered during one full volume is equal to 1/N of the prescribed dose, where N was the number of rescans (14). An alternative approach is called iso-layered repainting, in which first delivered several rescans within one energy plane before switching to the next plane with the dose per spot being limited by a maximal MU value (17).

The concept of spot-scanning arc therapy (SPArc) technique was introduced in 2016 to improve the dosimetric plan quality, robustness, and delivery efficiency of proton beam therapy. The technique demonstrated potential clinical benefits in several disease sites or indications (18–23). Whether this novel technique has any potential clinical benefits in the management of stage I non-small cell lung cancer and whether it is robust enough to be implemented in the hypo-fractionated lung SBRT has yet to be explored. Therefore, we proposed a comprehensive study is to 1) to build a lung SBRT model to evaluate the effectiveness of motion interplay mitigation *via* SPArc quantitatively; 2) to validate the model using clinical data sets and exploit the potential benefits.

## Materials and Methods

### *In Silico* 4D Interplay Phantom Model

Due to the target deformation, motion, and imaging artifact in the four-dimensional computed tomography (4DCT), it is challenging to analyze the interplay effect quantitatively using the patient dataset directly. Previous studies have suggested using a digital lung cancer phantom as a surrogate (24, 25). By introducing a digital phantom employed in a prior study, we built an in silico 4D interplay phantom model to mimic patient’s 4DCT datasets while eliminating the artifact and target deformation uncertainties (25). Since most lung tumor motion happens in the superior-inferior (SI) direction (26), a set of digital lung tumor phantoms 4DCT with different breathing induced motion amplitudes (5, 10, 15, and 20 mm in SI direction) were created (25, 27). The target was simulated using a sphere 3 cm in diameter with 1.0 g/cc density (28), close to the average target size measured in the patient group for this study. The gross tumor volume (GTV) was contoured on the lung window through the HU (Hounsfield unit) threshold at each phase image. The internal target volume (ITV) was generated by union GTVs at each phase.

#### Treatment Planning on the Phantom Model

5,000 cGy relative biological effectiveness [RBE] was prescribed to ITV in five fractions SBRT with RBE = 1.1 for proton plans and RBE = 1.0 for photon plans (29). Two-field IMPT plans were generated using the single field optimization (SFO) technique *via* lateral and posterior beams. SPArc plans were regenerated using a partial arc from 180 to 30° clockwise with a sampling frequency of 2.5°. Both planning strategies used the same robust optimization on average CT with ±5% range and 5 mm setup uncertainties corresponding to 21 scenarios in total with a 3 mm dose grid. The minimum monitor unit (MU) threshold per spot was 0.02 MU based on the IBA proton system (19, 23, 30, 31). Similar objective constraints for organs at risk (OARs) were used in both planning groups. All plans were normalized to guarantee 99% ITV was covered by the prescription dose. The SPArc optimization algorithm starts from a multi-field IMPT with coarse sampling frequency using the worst-case scenario robust optimization and gradually resample the control point to achieve a proton arc plan (18). The algorithm integrated the iterative approaches includes (A) control point re-sampling; (B) control point energy layers re-distribution; (C) energy layers filtration; (D) energy layers re-sampling; and (E) spot number reduction by filtration. Details of the algorithm are described by Ding and Li et al. in 2016 (18).

#### Interplay Effect Evaluation

The 4D dynamic dose was calculated to assess the interplay effect by synchronizing the breathing pattern with the simulated proton machine delivery sequence (19, 32). To calculate a single fraction 4D dynamic dose, the dose calculated on each phase image was accumulated via the deformable image registration to the reference phase (expiration end, phase 50%) (19, 32). Ten different starting phases were simulated based on a clinical 360-degree gantry machine parameter with one revolution per minute (RPM) gantry rotation speed, 2 ms spot position switching time, energy layer switching time (ELST) of 1 s, as well as a respiratory motion period of 4 s (19). GTV D99 was assessed along with target motion amplitude variation.

#### A Quantitative Interplay Effect Mitigation Evaluation

In this study, IMPT treatment delivery simulation using a volumetric repainting technique was denoted as IMPT_volumetric_, and IMPT using an iso-layered repainting technique denoted as IMPT_layer_. To quantitatively evaluate the effectiveness of interplay effect mitigation in lung SBRT, the single fraction 4D dynamic was compared between SPArc without repainting and IMPT_volumetric_, with different numbers of volumetric repainting (rescanning three, five, seven, and nine times respectively) and IMPT_layer_ with a series of maximum MU per spot (from 0.1 to 1.3 MU per spot).

### A Retrospective Dosimetric Planning Study

Ten patients with stage I NSCLC previously treated with volumetric modulated Arc therapy (VMAT) based SBRT at our institution were selected. All patients received 4DCT simulation using a helical CT scanner (Philips Brilliance Big Bore, Philips Healthcare System, Cleveland, OH). The GTVs and ITV were generated through the strategies described above as well. The patient characteristics, including tumor location, tumor size in diameter, and tumor motion, are listed in **Table 1**.

**Table 1:** Patient characteristics.

No.	Tumor Lobe	Tumor Size (mm)	Tumor Motion			
			SI (mm)	RL (mm)	PA (mm)	Offset (mm)
1	RUL	17.0	1.6	1.0	0.2	1.9
2	LUL	33.0	5.0	1.0	3.0	5.9
3	LLL	19.0	5.0	3.0	1.0	5.9
4	LLL	13.0	5.0	2.0	3.0	6.2
5	LUL	22.0	4.0	3.0	4.0	6.4
6	RUL	30.0	5.0	3.0	3.0	6.6
7	RUL	20.0	7.0	4.0	4.0	9.0
8	RUL	32.0	7.0	4.0	5.0	9.5
9	LLL	20.0	10.0	1.0	3.0	10.5
10	LLL	32.0	22.0	4.7	1.9	22.6

SI, superior inferior; RL, right left; PA, posterior anterior; RUL, right upper lobe; LUL, left upper lobe; LLL, left lower lobe; RLL, right lower lobe. Offset = (SI^2^ + Rl^2^ + PA^2^)^1/2^.

#### Treatment Planning in the Patient Dataset

The VMAT plans were generated using two to four partial arcs (control point frequency as 4°) based on the Elekta HD with 6 MV. The VMAT plan optimization starts from a coarse sampling of gantry position. New sample was added to achieve the desired sampling frequency, in which the Multileaf collimator (MLC) was linearly and gradually interpolated by the adjacent samples (33).

Two-field IMPT and SPArc with partial arc plans were generated, respectively. The prescription dose 5,000 cGy [RBE] was prescribed to 99% of the ITV. For a fair comparison between photon technique and proton technique, robust optimization-based ITV was used considering the same setup uncertainties as 5 mm, but ±5% range uncertainty was considered in proton planning.

#### Dosimetric Plan Quality Evaluation

The plan quality was evaluated based on the dose–volume histograms (DVHs) of target volume and OARs in the nominal plans. More specifically, all plans were compared for target coverage using conformity index (CI, the target volume covered by RX/the volume covered by RX). Dosimetric index for normal tissue sparing or OARs such as the Dmax or D0.1cc for the spinal cord, ribs and esophagus, Dmean for heart, ipsilateral lung (excluding ITV) as well as chest wall (CW) V30 (the volume received 3,000 cGy [RBE]) were evaluated by comparing SPArc planning group to VMAT and IMPT group.

The integral dose (ID) of radiation delivered to the whole patient body structure or external contour was analyzed (34). The ID definition was as following:

(1) $$\text{ID}(\text{Gy}\cdot \text{L})=\bar{D}(\text{Gy})\cdot \text{V}(\text{L})$$

where (Gy) is the mean dose delivered to volume V (L) (where L—liter).

#### Patient-Specific Interplay Effect Evaluation

Each case’s interplay effect was evaluated based on the 4D dynamic dose accumulation method mentioned in *Interplay Effect Evaluation* (19, 32).

#### Potential Clinical Benefit in Chestwall and Ipsilateral Lung Protection

Late chest wall toxicity after SBRT has been evaluated among three treatment modalities in this study. The probability of chest wall toxicity was calculated based on the odds ratios using the dosimetric parameter chest wall V30 (the volume of chest wall receiving 30 Gy) (35):

(2) $$Probability\;of\;CW\;toxicity\;b\text{y}\;V30(cc)=\frac{e\left[-3.151+\left(0.042*V30\right)\right]}{1+e\left[-3.151+\left(0.042*V30\right)\right]}$$

The incident of radiation pneumonitis for an ipsilateral lung was calculated based on the Lyman–Kutcher–Burman (LKB) model as following (36):

(3) $$\text{NTCP}=\frac{1}{\sqrt{2\pi }}\int_{-\infty }^{\text{t}}e^{-\frac{x^{2}}{2}}dx$$

(4) $$t=\frac{D-{TD}_{50}}{m\times {TD}_{50}}$$

where *TD_50_* is the tolerance dose for a 50% complication probability for uniform doses to the organ, and *m* is a dimensionless parameter for determining the slope of the complication probability according to the dose curve. And *D* is the equivalent dose(EUD), which is the DVH to a single dose value, representing the uniform dose that results in the survival of an equal number of clonogens in a non–homogeneously irradiated tumor. It is defined with the formula as (36):

(5) $$\text{EUD}={\left(\sum_{i=1}^{N}v_{i}D_{i}^{a}\right)}^{\frac{1}{a}}$$

where *Di* is the dose for each bin in a differential DVH, *vi* is the volume in a specific dose bin *i*, and *N* is the unequal fractional sub-volume. The ‘*a*’ value is a parameter equal to *1/n*, in which *n* represents the volume dependence of the complication probability. The parameter set for the lung tissue were taken from Burman et al. (TD50 = 24.5 Gy, m = 0.18, and a = 0.87) (37, 38). According to the LQ model, the dose axis of the DVH was re-scaled to the equivalent dose in 2 Gy per fractions and the method described by Van den Heuvel with assuming α/β ratio of 3 Gy for the ipsilateral normal lung tissue (39, 40).

The dosimetric index from SPArc was utilized as a reference. By comparing with two other treatment technologies (IMPT and VMAT), the differences were assessed with a paired, 2-tailed non-parametric Wilcoxon signed-rank test via SPSS 21.0 software (International Business Machines, Armonk, New York), respectively, and p values less than 0.05 were considered statistically significant.

## Result

### *In Silico* 4D Interplay Phantom Model

#### The Interplay Effect Evaluation

**Figure 1:**
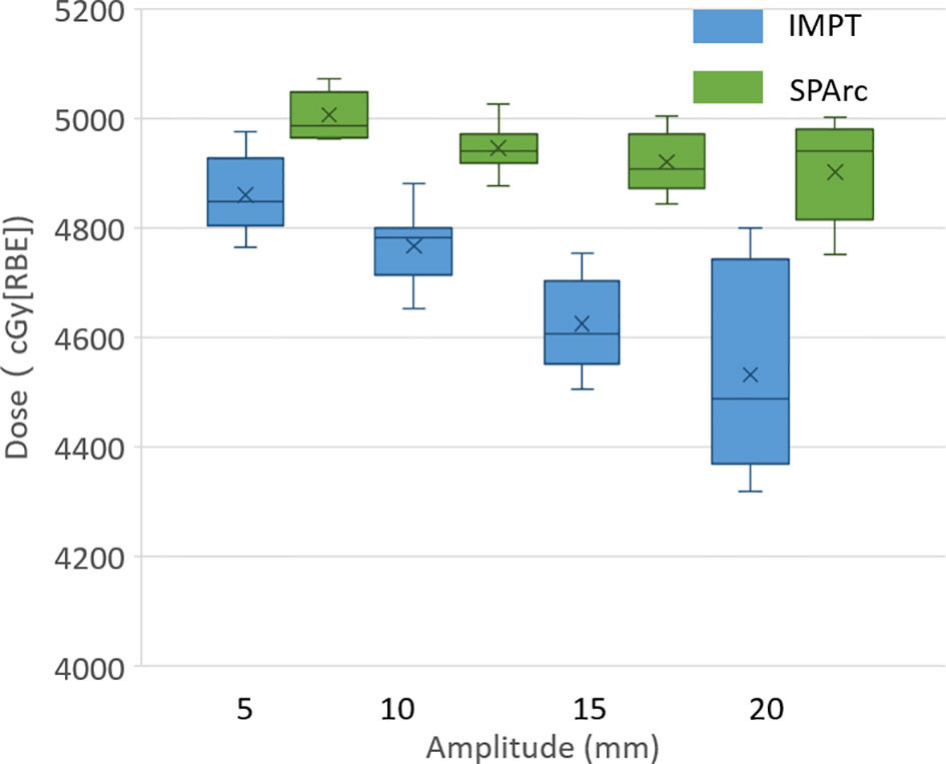
Single fraction dynamic dose for target D99 along with different motion amplitude from 5 to 20 mm.

The study showed that the dose to target degraded as the tumor motion amplitude increased in the IMPT planning, which was agreed with the previous reports (16, 41, 42). **Figure 1** displayed the target D99 in relationship with various motion amplitudes. SPArc could significantly improve the target coverage compared to IMPT through all the different motion amplitudes, even without any repainting. More specially, the average relative target D99 degradation *via* single fraction 4D dynamic dose accumulation were 2.51 vs 0.00% (p <0.01), 4.01 vs 0.10% (p <0.01), 6.61 vs 1.29% (p <0.01), 8.40 vs 1.70% (p <0.01) for IMPT vs SPArc at different breathing amplitude (5, 10, 15 and 20 mm), respectively.

#### Comparison of Mitigation Effectiveness in the Interplay Effect With Repainting IMPT

**Figure 2:**
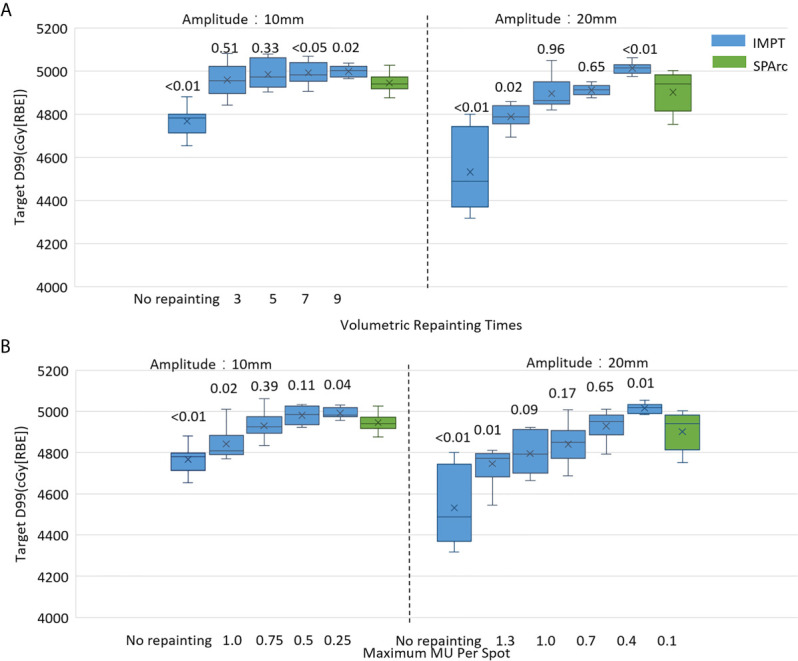
Single fraction 4D dynamic dose comparison between SPArc (green) and **(A)** different number of volumetric repainting times IMPT_volumetric,_ and **(B)** IMPTlayer with different maximum MU per spot.

The motion interplay effect can be compensated by increasing the total number of volumetric repainting or constraining the maximum MU per spot using iso-layered repainting. The single fraction 4D dynamic dose accumulation for target D99 with the number of volumetric repainting times and maximum MU per spot for the target motion 10 and 20 mm was displayed in **Figures 2A, B**, respectively. At 10 mm target motion amplitude, GTV D99 was 4,767 ± 63 cGy [RBE] (p <0.01) in IMPT without repainting, which is less than 4,950 ± 41 cGy [RBE] in SPArc. IMPT_volumetric_ increased GTV D99 to 4,959 ± 76 cGy [RBE] (p = 0.51) and 4,985 ± 66 cGy [RBE] (p = 0.33) with three and five times of volumetric repainting (**Figure 2A**); IMPT_layer_ increased GTV D99 to 4,931 ± 78 cGy [RBE] (p = 0.39) and 4,981 ± 66cGy [RBE] (p = 0.11) with maximum MU per spot as 0.75 and 0.50 MU respectively (**Figure 2B**), compared to SPArc. It is interesting to find that SPArc is as effective as three to five times of volumetric repainting IMPT or iso-layered repainting with maximum MU per spot as 0.75 to 0.5 MU at 10 mm target motion amplitude.

Moreover, in the target motion with 20 mm amplitude, GTV D99 was 4,532 ± 180 cGy [RBE] (IMPT without repainting) vs 4,902 ± 94 cGy [RBE] (SPArc) (p = 0.01). SPArc was as effective as five to seven times of volumetric repainting or iso-layered repainting with maximum MU per spot as 0.7 to 0.4 MU in IMPT for where GTV D99 received 4,896 ± 75 cGy [RBE] (p = 0.96) and 4,912 ± 26 cGy [RBE] (p = 0.65) for IMPT with volumetric repainting five and seven times, respectively (**Figure 2A**). Meanwhile, GTV D99 reached as 4,841 ± 102 cGy [RBE] (p = 0.09) and 4,929 ± 71 cGy [RBE] (p = 0.65) during IMPT_layer_ with maximum MU per spot of 0.7 and 0.4 MU (**Figure 2B**), respectively.

### Retrospective Study Using Patient Dataset

#### Plan Quality Evaluation

**Figure 3:**
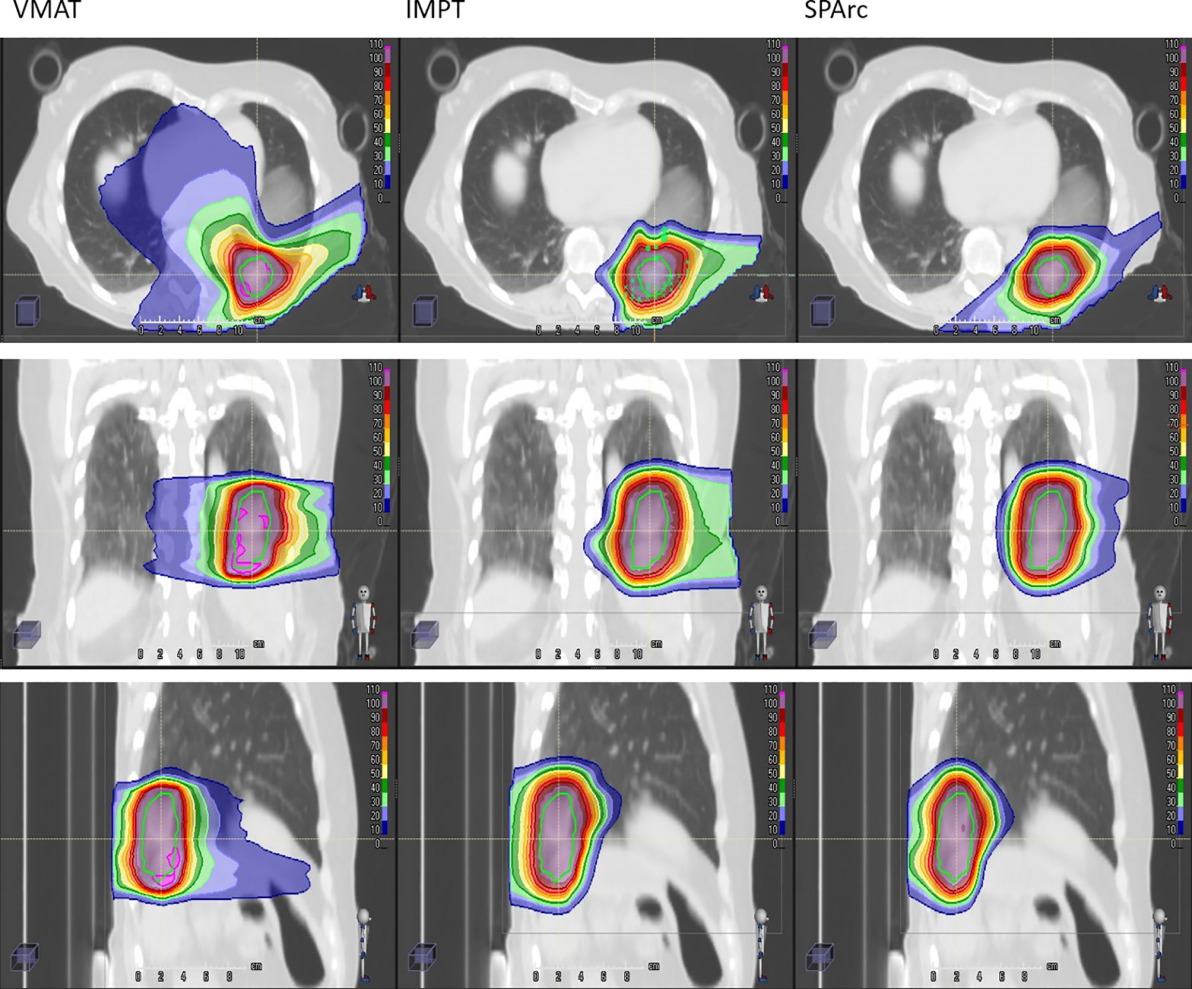
Isodose distributions of patient #10 for treatment plan using VMAT (first column), IMPT (the second column), and SPArc (the third column). The green contour represents ITV. 100% dose is equal to prescription dose.

Taking advantage of more degrees of freedom in the optimization through arc(s) trajectory, VMAT and SPArc planning groups demonstrated superior dose conformity to the target. Isodose distributions of patient #10 for treatment plan using VMAT (first column), IMPT (the second column), and SPArc (the third column) was displayed in **Figure 3**.

SPArc improved CI from 0.31 ± 0.08 in IMPT to 0.38 ± 0.10 (p = 0.01). This feature allows SPArc to spare more OARs such as spinal cord and ribs than IMPT. In addition, SPArc plans significantly reduced the Dmean of the ipsilateral lung from 503 ± 1 76 cGy [RBE] to 418 ± 140 cGy [RBE] (p = 0.01) and Dmax of ribs from 4,369 ± 978 cGy [RBE] to 4,151 ± 1,015 cGy [RBE] (p = 0.02) compared to IMPT respectively. V30 of the chest wall was significantly reduced from 30 ± 22 cc to 20 ± 14 cc (p = 0.02) compared to IMPT (**Table 2**).

**Table 2:** Dosimetry results for the three planning modalities.

	VMAT	SPArc	IMPT	p value VMAT vs SPArc	p value IMPT vs SPArc
Spinal Cord Dmax (cGy) [RBE]	1,026 ± 494	300 ± 530	338 ± 604	0.01	0.35
Ipsilateral lung Dmean (cGy) [RBE]	659 ± 200	418 ± 140	503 ± 176	0.01	0.01
Cheat Wall V30 (cc)	60 ± 37	20 ± 14	30 ± 22	<0.01	0.02
Heart Dmean (cGy) [RBE]	288 ± 253	8 ± 11	8 ± 9	0.01	0.83
Esophagus Dmax (cGy) [RBE]	1,611 ± 1,361	501 ± 1,565	541 ± 1,576	<0.01	0.16
Ribs Dmax (cGy) [RBE]	4,770 ± 1,059	4151 ± 1,015	4,369 ± 978	0.01	0.02
ID(Gy·L)	38.38 ± 17.10	16.13 ± 5.36	18.40 ± 5.79	0.01	0.01
CI	0.39 ± 0.12	0.38 ± 0.10	0.31 ± 0.08	0.54	0.01
The probability of CW toxicity(%)	40.2 ± 29.0	10.1 ± 5.4	16.3 ± 12.0	0.01	0.01

ID, integral dose; CW, chest wall.

Compared to VMAT, SPArc significantly reduced the dose to OARs listed in **Table 2**. More specially, SPArc significantly reduced maximum dose to spinal cord: 1,026 ± 494 cGy [RBE] (VMAT) vs 300 ± 530 cGy [RBE] (SPArc) (p = 0.01), esophagus: 1,611 ± 1,361 cGy [RBE] (VMAT) vs 501 ± 1,565 cGy [RBE] (SPArc) (p <0.01), ribs: 4,770 ± 1,059 cGy [RBE] (VMAT) vs 4,151 ± 1,015 cGy [RBE] (SPArc) (p = 0.01). In addition, SPArc significantly reduced the mean dose of the ipsilateral lung from 659 ± 200 cGy [RBE] (VMAT) to 418 ± 140 cGy [RBE] (SPArc) (p = 0.01), and the mean dose of heart from 288 ± 253 cGy [RBE] (VMAT) to 8 ± 11 cGy [RBE] (SPArc) (p = 0.01). The chest wall V30 was also significantly reduced *via* SPArc plan from 60 ± 37 cc to 20 ± 14 cc (p <0.01). The study also found that SPArc (16.13 ± 5.36 Gy·L) significantly reduced ID comparing to both IMPT (18.40 ± 5.79 Gy·L, p = 0.01) and VMAT (38.38 ± 17.10 Gy·L, p = 0.01) planning group.

#### Interplay Effect Evaluation in the Patient Population

**Figure 4:**
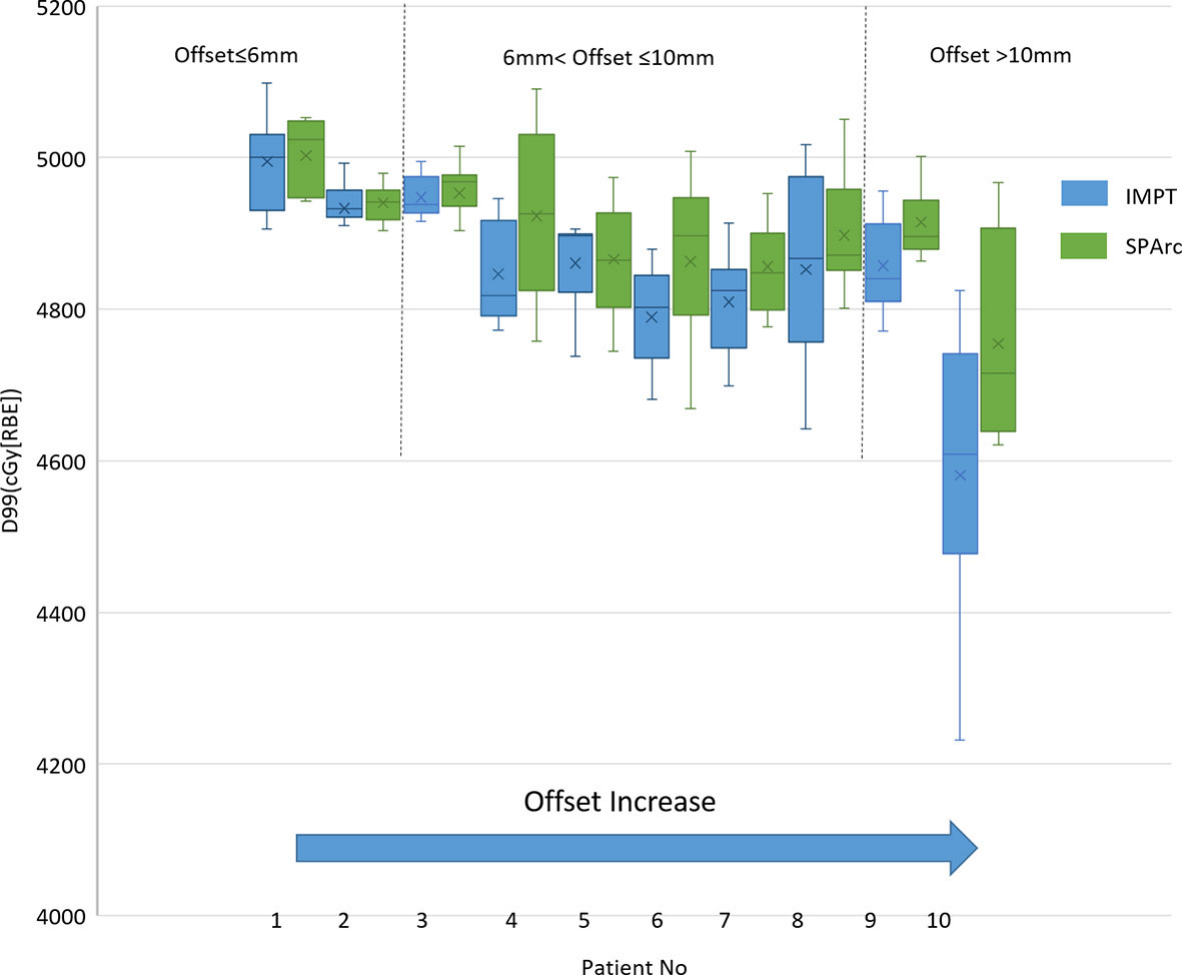
Single fraction dynamic dose for target coverage D99 for ten patients.

The single fraction 4D dynamic dose accumulation showed that target D99 was degraded due to the interplay effect in IMPT without repainting (**Figure 4**) among the ten patients. Similar to the 4D interplay phantom model, the retrospective study showed a similar trend of target dose coverage degradation with increased breathing-induced motion amplitudes. SPArc significantly mitigated interplay effect compared to IMPT among cases where breathing-induced motion cannot be ignored. Even though the target’s motion and shape were complicated as these parameters are patient-specific, the patient cases’ trend was consistent with the 4D interplay phantom study (Comparison of Mitigation Effectiveness in the Interplay Effect With Repainting IMPT) when the amplitudes of breathing induced motion is large (**Figure 4**). More specifically, the target D99 of 4D dynamic dose was 4,514 ± 138 cGy [RBE] (IMPT without repainting) vs 4,755 ± 129 cGy [RBE] (SPArc) (p = 0.01) fort the patient #10.

### The Probability of Chest Wall Toxicity and Radiation-Induced Pneumonitis

Due to SPArc significantly spared chest wall better than both IMPT and VMAT. The consequence clinic benefit was obvious, where the probability of CW toxicity were improved from 40.2 ± 29.0% (VMAT) (p = 0.01) and 16.3 ± 12.0 (IMPT) (p = 0.01) to 10.1 ± 5.4% (SPArc).

All three treatment modalities were able to spare ipsilateral lung tissue well. The corresponding incidence of radiation pneumonitis was fairly low, all of them were approximate to 0% on average.

## Discussion

This is the first study to explore the feasibility of using SPArc in hypo-fractionated treatment in mobile targets and its potential clinical benefits in lung SBRT. We quantify the effectiveness of using SPArc to mitigate the motion interplay effect using digital 4D lung cancer phantom and validate the model through a retrospective dosimetric study. The result confirmed the previous report that the interplay effect led to a deterioration of the dose distribution (16, 41, 42). The larger amplitude in motion, the more deterioration in the target coverage. Such a trend was consistent within the retrospective dosimetric study findings in the ten patient cases (**Figure 4**), even though the tumor shape, size, 3-dimentional tumor motion, and density variation during the breathing cycle were complicated for the patient population. For the patient group with 6 mm <target offset ≤10 mm, the target D99 degradation was 3.36 ± 0.55% on average for IMPT, and 2.05 ± 0.53% SPArc. Thus, it indicated that SPArc or IMPT with repainting was preferred to lung SBRT rather than IMPT alone without repainting. When the target offset >10 mm, the relative target dose D99 degradation was greater than 3% in both phantom and patient cases, indicating that IMPT poses a potential risk in missing part of the target in lung SBRT. In contrast, SPArc could mitigate the target dose degradation caused by interplay effect well in the 4D phantom model. The phantom study indicated that SPArc is as effective as five times of volumetric repainting IMPT in terms of interplay effect mitigation at 10 mm target motion amplitude. Only SI directional and rigid motion being considered in 4D phantom model, it required further investigation since 3D motion and complicated shape changes occurred for the clinical patients. Despite the effectiveness of motion interplay mitigation SPArc offered was compromised in the patient group, it was significantly superior to IMPT without repainting.

The previous study indicated that repainting might not be needed when multiple filed are applied with target amplitude up to 6 mm, because dose blurring effects appear negligible between standard delivery and repainting technique (43). A similar phenomenon was observed in our study as well, where the interplay effect between SPArc and IMPT is very close with target motion less than 6 mm. Additionally, Knopf et al. also demonstrated that IMPT with multiple beams was able to mitigate the interplay effect for targets with large motion amplitude (43), which was confirmed in our study. Our result indicated that SPArc plan could offer superior interplay effect mitigation through applying many beam angles via arc trajectory for the target motion more than10mm, compared to IMPT with two beams.

Lung SBRT for Stage I NSCLC is a highly effective treatment that is being increasingly utilized (4, 44, 45). Lung SBRT is characterized by using a hypo-fractionated treatment course with a biological equivalent dose of at least 100 Gy. Proton lung SBRT offers increased conformality compared to photon lung SBRT; however, there is uncertainty in tumor coverage mainly due to the interplay effect. Thus, proton lung SBRT commonly has been described with passive scattering techniques typically using at least ten fractions (46, 47). Chen et al. reported on using lung SBRT with IMPT with a few patients receiving eight fractions, although the majority had at least ten fractions (48). This study indicated that SPArc’s ability to mitigate the interplay effect could improve the normal tissue toxicity while also providing the means to use three to five fraction regimens commonly used with photon SBRT. Even single fractions of photon lung SBRT were shown in RTOG 0915 to have comparable efficacy and toxicity to four fractions (48, 49). The logistical and financial benefits of hypofractionation are attractive in this population of patients with multiple medical comorbidities. SPArc’s increased robustness enables the use.

**Figure 5:**
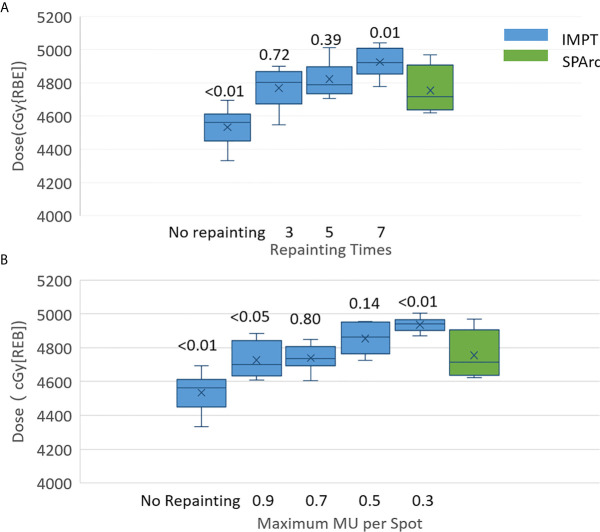
Single fraction 4D dynamic dose of patient #10 for target D99 along with **(A)** volumetric repainting times and **(B)** maximum MU per spot. The top value is the p value for the comparison of target D99 between IMPT with repainting and SPArc without repainting.

Other motion management strategies such as passive pressure technique, gating, the breath-hold approach could be implemented in the clinical practice, but these procedures prolong treatment time, which causes additional intra-fractionation motion or setup uncertainties (50). This study demonstrated that SPArc could effectively mitigate motion interplay. This new finding of utilizing SPArc to mitigate the interplay effect opens a new direction of motion management strategy by increase the degree of freedom such as arc(s) trajectory to effectively reduce the dosimetric impact from each beam’s direction. More specifically, for patient #10, where the breathing-induced motion exceeds 2 cm, the effectiveness of interplay effect mitigation of using SPArc technique reached five times of IMPT with volumetric repainting or IMPT using iso-layered repainting with maximum MU per spot as 0.5 MU at most, in which the corresponding GTV D99 were 4,822 ± 98 cGy [RBE] (p = 0.39) and 4,854 ± 86 cGy [RBE] (p = 0.14) (**Figures 5A, B**).

In general, SBRT is associated with a low incidence of acute and late toxicity. However, late chest well toxicity such as chest pain has been reported, typically mild to moderate. Moreover, chest wall pain commonly occurs with a median time of onset of greater than six months after the treatment. This study shows that SPArc has significantly spared the chest wall V30. Consequently, the probability of CW toxicity was improved considerably from 40.2 ± 29.0% (VMAT) (p = 0.01) and 16.3 ± 12.0% (IMPT) (P = 0.01) to 10.1 ± 5.4% (SPArc), which would improve the probability of chest wall toxicity and patient’s life quality.

## Conclusion

A 4D interplay digital phantom model for mobile lung target was established to evaluate the effectiveness of interplay effect mitigation quantitatively. SPArc, as a novel proton treatment technique, could significantly reduce the dosimetric impact from the interplay effect and potentially reduce the Chestwall pain in lung SBRT.

## Data Availability Statement

The raw data supporting the conclusions of this article will be made available by the authors, without undue reservation.

## Ethics Statement

The studies involving human participants were reviewed and approved by Beaumont Health Institutional Review Board. Written informed consent for participation was not required for this study in accordance with the national legislation and the institutional requirements.

## Author Contributions

GL contributed to the acquisition, analysis, and interpretation of data and drafted and designed the paper. XL provided technical support. IG and RD contributed to revising the paper and providing clinical inputs. AQ, DY, CS, and SZ provided support in imaging acquisition. LZ contributed to the statistical analysis. XD contributed to the design of the study, revised the draft, and led the research direction. All authors contributed to the article and approved the submitted version.

## Funding

The study is supported by Ion Beam Application S.A. (IBA, Belgium). The funder was not involved in the study design, collection, analysis, interpretation of data, the writing of this article or the decision to submit it for publication.

## Conflict of Interest

XD, XL, and DY have a patent related to spot-scanning arc therapy.

The remaining authors declare that the research was conducted in the absence of any commercial or financial relationships that could be construed as a potential conflict of interest.
